# Pulmonary Vein Anatomy, Cooling Kinetics, and Recurrence After Cryoballoon Ablation for Atrial Fibrillation

**DOI:** 10.1002/joa3.70511

**Published:** 2026-11-02

**Authors:** Kuo‐Ming Yang, Hsiang‐Chiang Hsiao, Tung‐Chen Yeh, Chun‐Wang Chiou, Jeng‐Fung Hung, Cheng‐Hung Chiang

**Affiliations:** ^1^ Cardiovascular Medical Center Kaohsiung Veterans General Hospital Kaohsiung Taiwan; ^2^ Department of Critical Care Medicine Kaohsiung Veterans General Hospital Kaohsiung Taiwan; ^3^ Department of Optometry Shu‐Zen Junior College of Medicine and Management Kaohsiung Taiwan; ^4^ School of Medicine, College of Medicine National Yang Ming Chiao Tung University Taipei Taiwan; ^5^ Institute of Science Education and Environmental Education National Kaohsiung Normal University Kaohsiung Taiwan; ^6^ Department of Physical Therapy Shu‐Zen Junior College of Medicine and Management Kaohsiung Taiwan

**Keywords:** atrial fibrillation, catheter ablation, cryosurgery, pulmonary veins, tomography X‐ray computed

## Abstract

**Background:**

Cryoballoon pulmonary vein isolation depends on balloon‐to‐ostium contact. We assessed whether computed tomography‐derived pulmonary vein ostial area (OA) and ovality index (OI) were associated with cooling after accounting for pulmonary vein (PV) branch and within‐patient clustering, and whether PV anatomy was associated with recurrence.

**Methods:**

We retrospectively studied 120 patients with symptomatic atrial fibrillation undergoing second‐generation cryoballoon ablation. Vein‐level associations with nadir temperature, 1 min temperature, and time to isolation were assessed using linear mixed‐effects models with a patient random intercept and PV branch as a fixed effect; generalized estimating equations served as sensitivity analyses. Given 23 post‐blanking recurrences, logistic models were limited to left atrial volume index (LAVI) and one clinically selected covariate, with Firth‐penalized regression as a sensitivity analysis.

**Results:**

All patients achieved acute PV isolation. Larger OA was associated with lower nadir temperature (*β* = −0.583°C per 50 mm^2^; 95% CI, −0.910 to −0.257; *p* < 0.001) and 1 min temperature (*β* = −0.441°C per 50 mm^2^; 95% CI, −0.764 to −0.118; *p* = 0.008) after branch and clustering adjustment. OI was not associated with either temperature outcome. Twenty‐three patients (19.2%) developed recurrent atrial arrhythmia. LAVI was associated with recurrence (adjusted OR, 1.659 per 10 mL/m^2^; 95% CI, 1.250–2.201; *p* < 0.001), whereas persistent AF was not.

**Conclusions:**

OA showed a modest association with cryoballoon temperatures but should not be used as a stand‐alone prognostic marker. LAVI remained associated with recurrence in parsimonious patient‐level analyses.

## Graphical abstract

After adjustment for pulmonary vein branch and within‐patient clustering, larger ostial area was associated with modestly lower cryoballoon temperatures. Larger left atrial volume index was associated with atrial arrhythmia recurrence in patient‐level models.

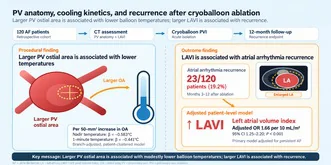

## 1 Introduction

Atrial fibrillation (AF) is the most common sustained arrhythmia worldwide and is associated with substantial morbidity, including stroke and heart failure [1, 2]. Catheter ablation is an established rhythm‐control strategy for symptomatic AF and is superior to antiarrhythmic drug therapy for maintaining sinus rhythm in appropriately selected patients [3]. Cryoballoon ablation (CBA) is a widely adopted approach for pulmonary vein isolation (PVI) and provides outcomes comparable to point‐by‐point radiofrequency ablation [4]. Nevertheless, recurrent atrial arrhythmia still occurs in a clinically meaningful proportion of patients after apparently successful cryoballoon PVI [5, 6, 7].

Effective PVI with CBA depends on stable balloon‐to‐ostium contact and complete pulmonary vein occlusion during energy delivery [8]. Pulmonary vein (PV) anatomy may therefore influence balloon occlusion and cryokinetic behavior during energy delivery [9, 10, 11, 12]. Larger PV dimensions or ostial area have been associated with lower nadir temperatures [11, 12]. Greater ovality may make complete balloon occlusion more difficult [9, 10].

Prior studies evaluating the relationship between PV anatomy and cryoballoon performance have yielded mixed results. Some studies have suggested that PV size and geometry influence cryokinetic behavior or procedural success [9, 11, 12, 13]. In contrast, other studies have found little or no effect of PV ovality on acute procedural outcomes [14]. Recurrence after AF ablation is multifactorial and may reflect PV reconnection, non‐PV triggers, or progressive atrial remodeling driven by patient‐level substrate [15, 16, 17].

The clinical utility of detailed preprocedural CT measurement remains uncertain. If PV anatomy affects intraprocedural cooling but does not predict late recurrence, its value would lie mainly in procedural planning, such as anticipating branch‐specific occlusion or cooling behavior, rather than in long‐term risk stratification. Importantly, individual pulmonary veins are nested within patients, and the four major branches differ systematically in size, orientation, and cooling behavior. The present study therefore evaluated CT‐derived ostial area and ovality using patient‐clustered, branch‐adjusted models for cryokinetic endpoints, and separately examined whether PV anatomy adds prognostic information beyond patient‐level atrial substrate, particularly left atrial volume index (LAVI).

## 2 Methods

### 2.1 Study Population

This retrospective single‐center study included adult patients (> 18 years) with symptomatic AF who underwent CBA between January 2021 and December 2023. All patients had AF that was refractory to or intolerant of at least one antiarrhythmic medication before ablation. Baseline characteristics, including age, sex, AF type, cardiovascular comorbidities, and CHA_2_DS_2_‐VASc score, were recorded for all participants. The study protocol was approved by the Institutional Ethics Committee of our hospital, and the requirement for individual informed consent was waived because of the retrospective design.

### 2.2 Pre‐Ablation CT Imaging and PV Anatomy Analysis

All patients underwent a pre‐ablation cardiac CT scan to delineate the left atrium (LA) and PV anatomy. Imaging was performed using a 256‐slice multidetector CT scanner with an optimized protocol for pulmonary venous phase imaging. An 80–100 mL bolus of iodinated contrast was injected at 4 mL/s, followed by a 40 mL saline flush. Scanning was triggered after a brief delay at peak LA opacification to capture high‐resolution images of the LA and PVs (slice thickness of 0.625 mm).

The acquired CT images were post‐processed to reconstruct a three‐dimensional model of the LA and PVs. Using an electroanatomic mapping software platform (EnSite Precision, St. Jude Medical, St. Paul, MN, USA), the ostium of each PV was identified on the reconstructed model. For each PV, the maximal diameter (Dmax) and minimal diameter (Dmin) at the ostium were measured (Figure 1). The PV ovality index (OI) was defined as Dmax/Dmin, and the PV ostial area (OA) was calculated using the formula for an ellipse: OA = (Dmax × Dmin × π)/4. The calculated OA was treated as an estimate of ostial cross‐sectional area. LA volume was assessed using preprocedural CT. The LAVI was calculated by dividing LA volume by body surface area and expressed in mL/m^2^.

**FIGURE 1:**
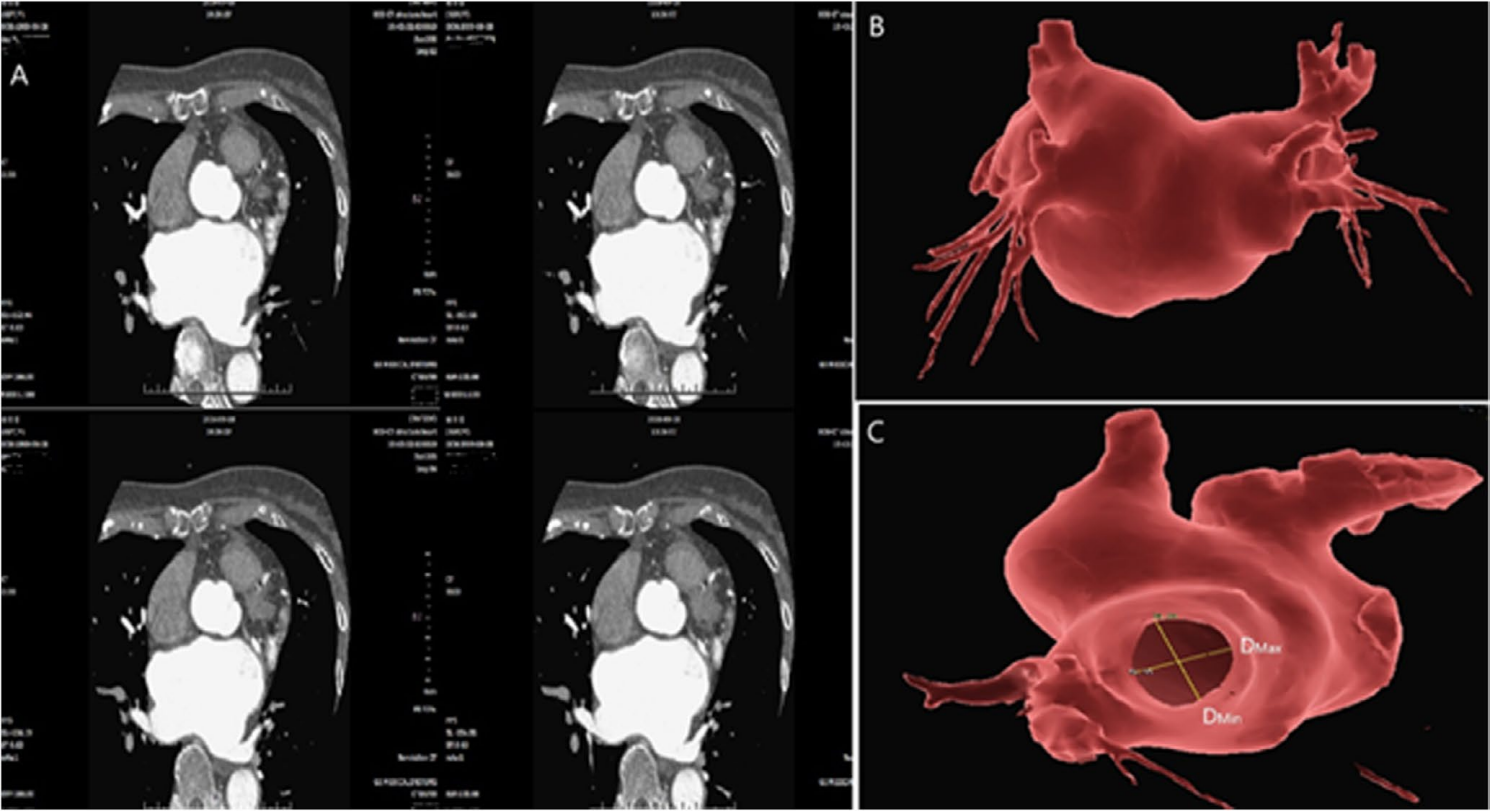
Computed tomography (CT) scan and reconstruction of the left atrium (LA) and pulmonary veins (PVs) for structural assessment before cryoablation. (A) Pre‐procedural CT scan. (B) Reconstruction of the LA and PVs using the EnSite Precision system. (C) Measurement of the maximal diameter (Dmax) and minimal diameter (Dmin) of the PV ostium.

Four patients had a left common pulmonary vein (LCPV). In these patients, the LCPV was treated as a single anatomical unit, with ostial measurements obtained at the common ostium. For the vein‐level analyses, each LCPV contributed one observation and was assigned to the LSPV category, with no separate LIPV observation. Consequently, a total of 476 pulmonary venous anatomical units were included in the analyses.

### 2.3 Cryoballoon Ablation Procedure

All ablation procedures were performed under conscious sedation. After venous access, a single transseptal puncture (using a BRK‐1 needle, Abbott, St. Paul, MN, USA) was performed under fluoroscopic guidance to access the LA. A 28 mm second‐generation cryoballoon catheter (Arctic Front Advance, Medtronic, Minneapolis, MN, USA) was advanced into the LA via a transseptal sheath (FlexCath Advance Steerable Sheath, Medtronic, Minneapolis, MN, USA). An inner lumen circular mapping catheter (Achieve Mapping Catheters, Medtronic, Minneapolis, MN, USA) was inserted into each PV to help position the cryoballoon at the PV ostium.

Proper occlusion of each PV by the balloon was confirmed with selective contrast injection before freezing. The usual ablation sequence was left superior PV (LSPV), left inferior PV (LIPV), right inferior PV (RIPV), and right superior PV (RSPV). Each PV underwent two cryoapplications when achievable, each lasting 120–180 s. During each application, time to isolation (TTI), temperature at 1 min, and nadir temperature were recorded when available. Applications were terminated early for safety if phrenic nerve palsy occurred during right‐sided ablation or if the balloon temperature fell below −60°C. Additional freeze applications were delivered when necessary, and focal radiofrequency touch‐up ablation was used only rarely when persistent gaps remained. Complete bidirectional PVI was confirmed for all PVs at the end of the procedure.

Because multiple cryoapplications could be delivered to each pulmonary venous anatomical unit, one cryoapplication was selected for each outcome analysis. When real‐time PV potentials were available, the application during which electrical isolation was first observed was selected, and nadir temperature, 1 min temperature, and TTI were obtained from that application. When real‐time isolation timing could not be determined but PVI was confirmed after a cryoapplication, the first application after which PVI was confirmed was selected. Nadir temperature and 1 min temperature were obtained from the application, whereas TTI was recorded as unavailable. All temperature measurements for a given pulmonary venous anatomical unit were derived from the same selected application. Temperature values below −60°C were recorded as −60°C. PVs with an unavailable measurement were excluded only from the corresponding outcome analysis, and no missing values were imputed.

### 2.4 Outcome Definitions and Follow‐Up

After ablation, patients generally continued antiarrhythmic medication for an empiric 3‐month blanking period to reduce early recurrences. Decisions regarding antiarrhythmic therapy beyond the blanking period were left to the treating physician. Follow‐up visits were scheduled at 1, 2, 3, 6, 9, and 12 months after the procedure, with additional visits as clinically indicated. Patients underwent clinical assessments and a 12‐lead electrocardiogram at each follow‐up.

Arrhythmia recurrence was defined as any documented atrial tachyarrhythmia (AF, atrial flutter, or atrial tachycardia) lasting at least 30 s between 3 and 12 months after the index ablation. Episodes occurring within the first 3 months were considered blanking‐period events and were not counted as recurrence. Management of recurrent arrhythmia, including consideration of repeat ablation, followed routine clinical practice.

### 2.5 Relationship of PV Anatomy to Cryokinetics

To evaluate the effect of PV anatomy on cryoballoon performance, individual PVs were analyzed at the vein level while preserving their nesting within patients. The primary anatomical exposures were OA, scaled per 50 mm^2^, and OI, scaled per 0.1 unit. PV branch was categorized as LSPV, LIPV, RSPV, or RIPV. Binary thresholds (OA ≥ 200 mm^2^ and OI ≥ 1.4) were retained only for descriptive summaries and graphical presentation; they were not used for the primary inferential analyses. The cryokinetic outcomes were temperature at 1 min, nadir temperature, and time to isolation. For recurrence analyses, PV measurements were summarized at the patient level as mean OA and mean OI.

### 2.6 Statistical Analysis

Continuous variables are presented as mean ± standard deviation or median (interquartile range), as appropriate, and categorical variables as counts and percentages. Branch‐stratified descriptive comparisons are shown to illustrate PV heterogeneity. Primary inference for cryokinetic outcomes was based on linear mixed‐effects models with a random intercept for patient to account for within‐patient clustering and fixed effects for PV branch. OA and OI were modeled as continuous variables using the scales described above. The primary model for each cryokinetic outcome included OA, OI, and PV branch. Generalized estimating equation models with an exchangeable working correlation structure and robust standard errors were used as sensitivity analyses. An OA‐by‐branch interaction was tested separately for each cryokinetic outcome to assess whether the OA association varied among PV branches.

For 1‐year post‐blanking recurrence, analyses were performed at the patient level. Univariable logistic regression was used for descriptive context only. Because there were 23 recurrence events, the revised multivariable strategy was deliberately parsimonious to reduce overfitting. The primary model included LAVI, scaled per 10 mL/m^2^, and persistent AF based on their established clinical relevance to arrhythmia recurrence. Sensitivity models replaced persistent AF with hypertension or mean PV OA to assess the stability of the LAVI estimate. Firth‐penalized logistic regression was performed as an additional sensitivity analysis. Stepwise selection and simultaneous adjustment for all candidate predictors were not used. Results are reported as regression coefficients or odds ratios with 95% confidence intervals. A two‐sided *p* < 0.05 was considered statistically significant. Statistical analyses were performed using SAS software (version 9.4, SAS Institute Inc., Cary, NC, USA).

## 3 Results

### 3.1 Patient Characteristics

A total of 120 patients who underwent CBA for AF were included. Baseline characteristics, cardiac measurements, and comorbidities are summarized in Table 1. The mean age was 58.63 ± 9.69 years (range 28–77), and 83.33% of the cohort were male. Paroxysmal AF was present in 102 patients (85%), whereas 18 patients (15%) had persistent AF. The mean CHA_2_DS_2_‐VASc score was 1.51 ± 1.54. The mean LAVI was 67.49 ± 20.58 mL/m^2^, and the mean left ventricular ejection fraction was 59.03% ± 4.13%. Common comorbidities included hypertension in 44 patients (36.67%), diabetes mellitus in 16 (13.33%), and heart failure in 16 (13.33%).

**TABLE 1:** Baseline clinical characteristics of the study population.

Basic characteristics	*n* = 120
Sex, female	20 (16.67%)
Age, years	58.63 ± 9.69
BMI, kg/m^2^	25.69 ± 3.53
LA volume, mL	118.35 ± 36.04
LAVI, mL/m^2^	67.49 ± 20.58
LVEF, %	59.03 ± 4.13
Persistent AF	18 (15.00%)
Hypertension	44 (36.67%)
Diabetes mellitus	16 (13.33%)
Coronary artery disease	24 (20.00%)
Congestive heart failure	16 (13.33%)
Prior stroke	10 (8.33%)
CHA_2_DS_2_‐VASc score	1.51 ± 1.54
Mean OA, mm^2^	210.94 ± 50.39
Mean OI	1.41 ± 0.22

Abbreviations: AF, atrial fibrillation; BMI, body mass index; LA, left atrium; LAVI, left atrial volume index; LVEF, left ventricular ejection fraction; OA, ostial area; OI, ovality index.

Pre‐ablation CT assessment of PV anatomy showed considerable variability in PV size and shape. Across all PVs, the mean calculated PV OA was 210.94 ± 50.39 mm^2^ and the mean PV OI was 1.41 ± 0.22.

### 3.2 Cryoablation Metrics and PV Anatomy

During cryoballoon ablation, all 120 patients achieved acute isolation of all targeted PVs. Among the 120 patients, four had a left common PV, resulting in 476 distinct pulmonary venous anatomical units available for vein‐level analysis. Nadir temperature and 1 min temperature were available for all 476 units, whereas TTI was available for 361 units. Outcome‐ and branch‐specific data availability is presented in Table S1.

Unadjusted branch‐stratified comparisons showed that the apparent association between larger OA and lower temperatures was not uniform across PV branches. The unadjusted association was strongest in the RSPV, whereas differences were smaller or absent in the LSPV, LIPV, and RIPV, indicating that pooled vein‐level comparisons could be confounded by branch.

In the primary mixed‐effects model accounting for patient clustering and PV branch, larger OA was associated with lower nadir temperature (*β* = −0.583°C per 50 mm^2^; 95% CI, −0.910 to −0.257; *p* < 0.001) and lower temperature at 1 min (*β* = −0.441°C per 50 mm^2^; 95% CI, −0.764 to −0.118; *p* = 0.008). OI was not associated with nadir temperature (*β* = 0.077°C per 0.1; 95% CI, −0.066 to –0.221; *p* = 0.290) or 1 min temperature (*β* = 0.119°C per 0.1; 95% CI, −0.022 to –0.261; *p* = 0.098). OA was not associated with log‐transformed TTI (*β* = −0.017 per 50 mm^2^; 95% CI, −0.057 to –0.023; *p* = 0.404). PV branch was associated with nadir temperature (overall *p* < 0.001). The OA‐by‐PV branch interaction *p*‐values were 0.999 for nadir temperature, 0.652 for 1 min temperature, and 0.585 for log‐transformed TTI, respectively. Branch‐specific OA estimates with 95% confidence intervals are presented in Table S2. GEE sensitivity analyses yielded similar estimates (Table 2). The adjusted branch‐stratified relationship between OA and nadir temperature is shown in Figure 2.

**TABLE 2:** Mixed‐effects and GEE models evaluating associations between pulmonary vein anatomy and cryokinetic outcomes.

Outcome	Model term	Mixed‐effects estimate (95% CI)	*p*	GEE estimate (95% CI)	*p*
Nadir temperature, °C	OA per 50 mm^2^	−0.583 (−0.910 to −0.257)	< 0.001	−0.583 (−0.899 to −0.267)	< 0.001
Nadir temperature, °C	OI per 0.1	0.077 (−0.066–0.221)	0.290	0.077 (−0.048–0.202)	0.226
Nadir temperature, °C	PV branch, overall		< 0.001		< 0.001
Nadir temperature, °C	OA × PV branch, overall		0.999		0.998
Temperature at 1 min, °C	OA per 50 mm^2^	−0.441 (−0.764 to −0.118)	0.008	−0.440 (−0.790 to −0.091)	0.014
Temperature at 1 min, °C	OI per 0.1	0.119 (−0.022–0.261)	0.098	0.119 (−0.008–0.246)	0.065
Temperature at 1 min, °C	PV branch, overall		< 0.001		< 0.001
Temperature at 1 min, °C	OA × PV branch, overall		0.652		0.571
log TTI	OA per 50 mm^2^	−0.017 (−0.057–0.023)	0.404	−0.017 (−0.060–0.026)	0.435
log TTI	OI per 0.1	0.013 (−0.006–0.032)	0.185	0.013 (−0.005–0.031)	0.160
log TTI	PV branch, overall		0.002		< 0.001
log TTI	OA × PV branch, overall		0.585		0.689

Abbreviations: CI, confidence interval; GEE, generalized estimating equation; log TTI, log‐transformed time to isolation; OA, ostial area; OI, ovality index; PV, pulmonary vein.

**FIGURE 2:**
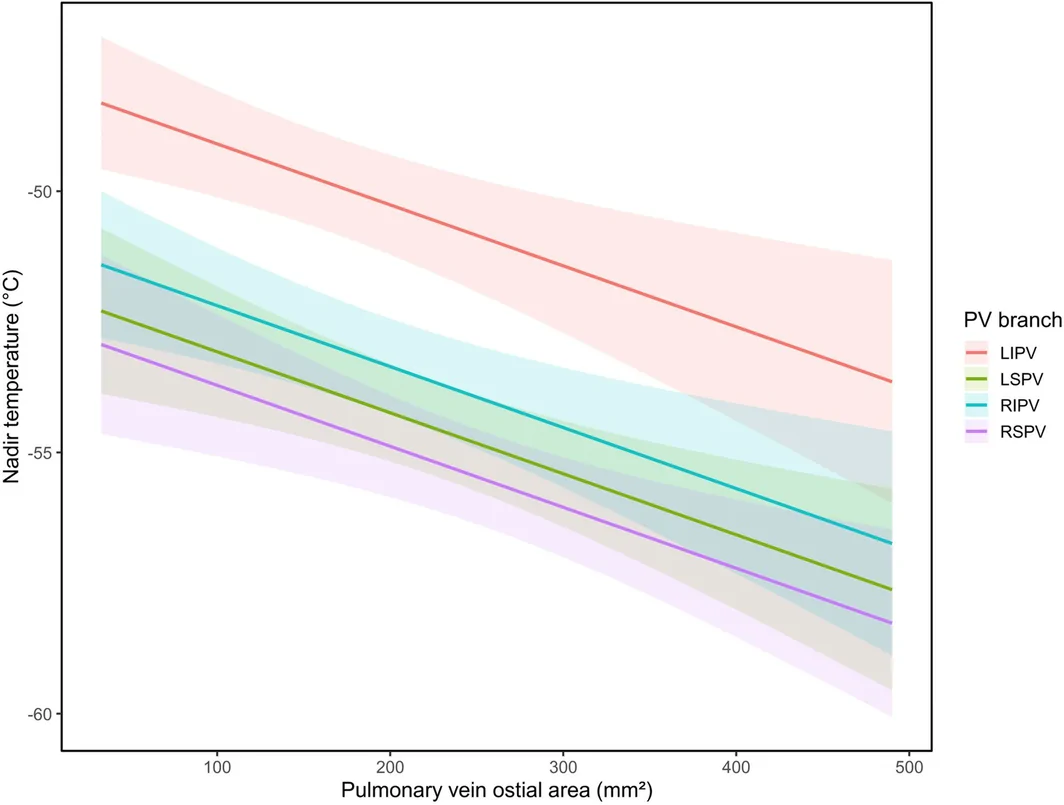
Branch‐stratified relationship between pulmonary vein ostial area (OA) and nadir temperature. The lines and corresponding shaded regions display the adjusted marginal estimates and 95% confidence intervals (CIs) from the mixed‐effects model adjusted for each PV branch and within‐patient clustering.

### 3.3 One‐Year Outcomes and Predictors of Arrhythmia Recurrence

All patients completed at least 12 months of follow‐up after the index ablation. During the 3‐month blanking period, some patients experienced transient episodes of AF or atrial flutter that were managed medically and were not classified as recurrence. After the blanking period, 23 of 120 patients (19.2%) developed recurrent atrial arrhythmia between months 3 and 12 after ablation.

Selected parsimonious recurrence models are summarized in Table 3. In univariable analyses, hypertension, persistent AF, LAVI, and mean PV OA were associated with recurrence; these findings were used for descriptive context and not for data‐driven multivariable selection.

**TABLE 3:** Associations of LAVI and selected clinical or anatomical covariates with 1 year atrial arrhythmia recurrence.

Model	Predictor	Adjusted OR (95% CI)	*p*
Primary logistic model	LAVI per 10 mL/m^2^	1.659 (1.250–2.201)	< 0.001
Primary logistic model	Persistent AF	1.827 (0.511–6.524)	0.354
Firth‐penalized sensitivity	LAVI per 10 mL/m^2^	1.616 (1.248–2.170)	< 0.001
Firth‐penalized sensitivity	Persistent AF	1.848 (0.511–6.210)	0.338
Sensitivity: LAVI + hypertension	LAVI per 10 mL/m^2^	1.695 (1.284–2.238)	< 0.001
Sensitivity: LAVI + hypertension	Hypertension	1.370 (0.478–3.926)	0.558
Sensitivity: LAVI + mean OA	LAVI per 10 mL/m^2^	1.709 (1.282–2.277)	< 0.001
Sensitivity: LAVI + mean OA	Mean OA per 50 mm^2^	1.080 (0.631–1.848)	0.780

Abbreviations: AF, atrial fibrillation; CI, confidence interval; LAVI, left atrial volume index; OA, ostial area; OR, odds ratio.

Because only 23 recurrence events occurred, the multivariable analysis was restricted to parsimonious models. In the primary model including LAVI and persistent AF, LAVI was associated with recurrence (adjusted OR, 1.659 per 10 mL/m^2^; 95% CI, 1.250–2.201; *p* < 0.001), whereas persistent AF was not (adjusted OR, 1.827; 95% CI, 0.511–6.524; *p* = 0.354) (Figure 3).

**FIGURE 3:**
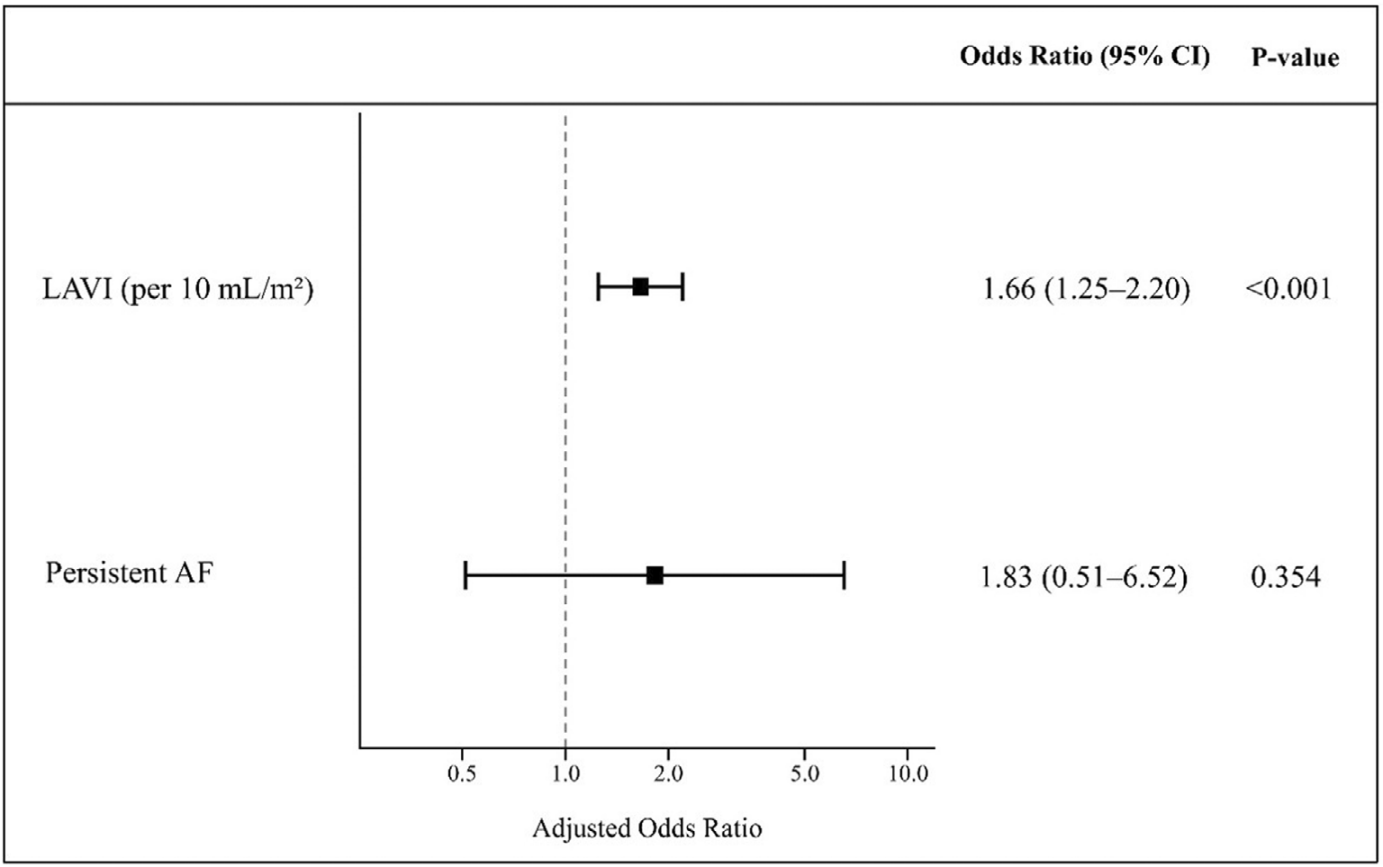
Forest plot showing adjusted odds ratios and 95% confidence intervals (CIs) from the prespecified parsimonious recurrence model. AF, atrial fibrillation; LAVI, left atrial volume index.

Firth‐penalized logistic regression produced a similar estimate for LAVI (adjusted OR, 1.616 per 10 mL/m^2^; 95% CI, 1.248–2.170; *p* < 0.001). In sensitivity models replacing persistent AF with hypertension or mean PV OA, the LAVI estimates remained similar (Table 3).

Across the parsimonious models examined, LAVI remained consistently associated with atrial arrhythmia recurrence. These analyses were intended to evaluate the stability of the LAVI association and should not be interpreted as development of a comprehensive prediction model.

## 4 Discussion

After accounting for within‐patient clustering and PV branch heterogeneity, this study distinguishes between anatomical correlates of cryoballoon temperature behavior and patient‐level factors associated with late clinical outcome. The principal findings were that: (1) PV branch was an important source of heterogeneity in vein‐level analyses; (2) Larger OA remained associated with modestly lower nadir and 1 min temperatures after branch and clustering adjustment; (3) OI was not independently associated with temperature outcomes; and (4). LAVI remained associated with 1‐year recurrence across parsimonious patient‐level models. These findings support a procedural‐versus‐prognostic interpretation of CT‐derived measurements without establishing a new clinical risk model.

### 4.1 PV Ostial Area and Acute Cryoballoon Performance

Consistent with prior studies reporting associations between PV anatomy and cryokinetic behavior, our unadjusted pooled vein‐level analysis suggested that larger OA was associated with lower 1 min and nadir temperatures [11, 12, 18]. Branch‐stratified findings, however, indicated that part of this association could reflect systematic anatomical and procedural differences among the LSPV, LIPV, RSPV, and RIPV. In mixed‐effects and GEE analyses that accounted for patient clustering and adjusted for PV branch, OA remained associated with modestly lower temperatures. The estimated difference was approximately 0.58°C in nadir temperature per 50 mm^2^ increase in OA. No significant OA‐by‐PV branch interaction was observed for nadir temperature, 1 min temperature, or TTI, suggesting no statistical evidence that the association between OA and these cryokinetic outcomes differed across PV branches. However, the branch‐specific estimates were relatively imprecise, and the absence of a statistically significant interaction should not be interpreted as definitive evidence of identical OA effects across all PV branches. Furthermore, this study did not test whether OA‐guided procedural modifications improved occlusion, reduced the number of applications, shortened procedure time, or increased lesion durability. Quantitative OA may therefore help explain variation in temperature behavior, but the present data do not establish a need for routine manual OA measurement solely to guide ablation strategy.

### 4.2 PV Ovality and Procedural Effectiveness

The effect of PV ovality on cryoballoon performance has remained controversial. Highly oval ostia may theoretically impair circumferential contact and promote temperature heterogeneity, and some prior studies suggested more challenging occlusion or less favorable cryokinetics in these veins [9, 10, 11, 12].

In the adjusted models, OI was not associated with nadir temperature (*β* = 0.077°C per 0.1; 95% CI, −0.066 to −0.221; *p* = 0.290) or 1 min temperature (*β* = 0.119°C per 0.1; 95% CI, −0.022 to –0.261; *p* = 0.098). This finding should be interpreted cautiously because ovality is a simplified two‐dimensional descriptor and may not fully represent the three‐dimensional shape of the ostium or the dynamic contact surface during balloon inflation. Contemporary cryoballoon systems may compensate for moderate anatomical variability when complete occlusion is achieved through careful positioning, contrast confirmation, and repositioning maneuvers.

Accordingly, PV ovality should be viewed primarily as a procedural consideration. It may alert the operator to potential challenges in achieving coaxial contact, but it should not be overinterpreted as a marker of long‐term clinical prognosis.

### 4.3 Clinical Substrate and One‐Year Recurrence

Recurrence after AF ablation is multifactorial and cannot be explained solely by acute PV geometry [15, 16, 17, 19, 20]. Although durable PVI remains the cornerstone of cryoballoon therapy, late arrhythmia recurrence may also reflect non‐PV triggers, residual atrial disease, and progressive structural remodeling. Our observed 1‐year recurrence rate of 19.2% falls within the range reported in contemporary cryoballoon series, although differences in rhythm‐monitoring intensity should be considered when comparing studies [5, 6, 7, 21].

The recurrence analysis was deliberately restricted because of the small number of outcome events. In the primary parsimonious model, LAVI was associated with recurrence (adjusted OR, 1.659 per 10 mL/m^2^; 95% CI, 1.250–2.201; *p* < 0.001). Previous studies have also reported an association between LAVI and AF recurrence following cryoballoon PVI, although the strength of this association may differ according to AF type [22, 23]. Left atrial enlargement reflects advanced atrial remodeling and may be associated with increased pressure burden and fibrotic substrate, thereby promoting recurrent arrhythmogenesis despite acute PVI. Persistent AF and hypertension remain clinically relevant indicators of advanced substrate, but their adjusted estimates should be interpreted cautiously in this modest cohort. The findings support assessment of atrial substrate and risk‐factor modification when counseling patients about expected ablation outcomes.

Mean PV OA was not associated with recurrence in the sensitivity model that also included LAVI. This finding is consistent with the concept that, once acute isolation is achieved, late recurrence may relate more to lesion durability, non‐PV triggers, and underlying atrial cardiomyopathy than to ostial geometry alone. Because the recurrence analysis was intentionally parsimonious, the results should not be presented as a comprehensive risk model or as evidence that LAVI is the only relevant prognostic factor.

### 4.4 Clinical Implications

The adjusted associations between OA and balloon temperature were modest, and PV anatomy was not shown to provide clinically useful long‐term risk stratification. These findings do not establish a need for routine manual quantification of PV OA solely for procedural decision‐making or prognosis. Preprocedural CT remains useful for defining overall LA and PV anatomy, whereas quantitative OA may primarily help explain branch‐related variation in balloon temperature. Counseling regarding long‐term rhythm outcome should place greater emphasis on patient‐level atrial substrate and modifiable risk factors.

### 4.5 Limitations

This study has several limitations. First, it was a retrospective single‐center analysis with a moderate sample size, and the results should be interpreted as hypothesis‐generating and validated in larger multicenter cohorts. Second, although mixed‐effects and GEE models addressed within‐patient clustering and PV branch, residual confounding by unmeasured procedural factors such as occlusion grade, sheath position, balloon alignment, freeze duration, and operator technique may remain. Third, OA was estimated from CT‐derived diameters using an elliptical formula and therefore cannot fully represent irregular ostial geometry or the dynamic balloon‐tissue contact surface. Fourth, TTI could be analyzed only when real‐time PV potentials were visible and the isolation moment could be identified. Therefore, the TTI analysis used an outcome‐specific available‐case sample and may be subject to selection related to signal visibility or procedural characteristics. Fifth, recurrence modeling was limited by only 23 outcome events; the multivariable analyses were deliberately parsimonious and should not be interpreted as a definitive prediction model. Sixth, repeat invasive mapping was not performed systematically in patients with recurrence, so the relative contributions of PV reconnection and non‐PV mechanisms could not be determined. Finally, follow‐up relied on scheduled visits and intermittent rhythm monitoring, which may have underestimated asymptomatic recurrence.

## 5 Conclusions

After accounting for PV branch and within‐patient clustering, larger PV OA was associated with modestly lower nadir and 1 min cryoballoon temperatures, whereas OI was not associated with these outcomes. In parsimonious patient‐level analyses, LAVI remained associated with atrial arrhythmia recurrence. These findings support a distinction between CT‐defined PV anatomy as a procedural characteristic and left atrial remodeling as a marker of long‐term rhythm outcome.

## Funding

The authors have nothing to report.

## Ethics Statement

This retrospective study was approved by the Institutional Ethics Committee of Kaohsiung Veterans General Hospital and was conducted in accordance with the Declaration of Helsinki. The requirement for individual informed consent was waived because of the retrospective study design. The IRB approval number is 24‐CT6‐10 (240508–1).

## Conflicts of Interest

The authors declare no conflicts of interest.

## Supporting information


**Table S1:** Outcome availability.

**Table S2:** Branch‐specific OA estimates.

## Data Availability

The data that support the findings of this study are available from the corresponding author upon reasonable request.
