# Supplementary material for: Pulmonary Vein Anatomy, Cooling Kinetics, and Recurrence After Cryoballoon Ablation for Atrial Fibrillation
Source: J Arrhythm. 2026 Nov 2;42(6):e70511. doi: 10.1002/joa3.70511 (PMC13728550; doi:10.1002/joa3.70511)
Supplement: Supplementary file 1 [file JOA3-42-e70511-s001.docx]

## Supplementary Table S1. Outcome Availability

| **Outcome** | **Branch** | **No. of cryoablation** | **Analyzed PVs** | **Patients** |
| --- | --- | --- | --- | --- |
| Nadir temperature | LSPV | 253 | 120 | 120 |
| Nadir temperature | LIPV | 240 | 116 | 116 |
| Nadir temperature | RSPV | 258 | 120 | 120 |
| Nadir temperature | RIPV | 255 | 120 | 120 |
| One-minute temperature | LSPV | 253 | 120 | 120 |
| One-minute temperature | LIPV | 240 | 116 | 116 |
| One-minute temperature | RSPV | 258 | 120 | 120 |
| One-minute temperature | RIPV | 255 | 120 | 120 |
| TTI | LSPV | 253 | 108 | 108 |
| TTI | LIPV | 240 | 89 | 89 |
| TTI | RSPV | 258 | 91 | 91 |
| TTI | RIPV | 255 | 73 | 73 |

Analyses used outcome-specific available cases without imputation. A PV could contribute to one outcome analysis even when another cryokinetic measurement was unavailable.

No. = number; LIPV = left inferior pulmonary vein; LSPV = left superior pulmonary vein; PV = pulmonary vein; PVs = pulmonary veins; RIPV = right inferior pulmonary vein; RSPV = right superior pulmonary vein; TTI = time to isolation.
