# Supplementary material for: Pulmonary Vein Anatomy, Cooling Kinetics, and Recurrence After Cryoballoon Ablation for Atrial Fibrillation
Source: J Arrhythm. 2026 Nov 2;42(6):e70511. doi: 10.1002/joa3.70511 (PMC13728550; doi:10.1002/joa3.70511)
Supplement: Supplementary file 2 [file JOA3-42-e70511-s002.docx]

**Supplementary Table S2.** Branch-Specific OA Estimates

| **Outcome** | **Branch** | **Analyzed PVs** | **Patients** | **OA effect per 50 mm²** | **95% CI** | **P** |
| --- | --- | --- | --- | --- | --- | --- |
| Nadir temperature | LSPV | 120 | 120 | -0.564 | -1.177 to 0.049 | 0.071 |
| Nadir temperature | LIPV | 116 | 116 | -0.552 | -1.440 to 0.336 | 0.223 |
| Nadir temperature | RSPV | 120 | 120 | -0.615 | -1.114 to -0.115 | 0.016 |
| Nadir temperature | RIPV | 120 | 120 | -0.566 | -1.175 to 0.042 | 0.068 |
| One-minute temperature | LSPV | 120 | 120 | -0.630 | -1.228 to -0.033 | 0.039 |
| One-minute temperature | LIPV | 116 | 116 | -0.001 | -0.867 to 0.865 | 0.998 |
| One-minute temperature | RSPV | 120 | 120 | -0.380 | -0.867 to 0.107 | 0.126 |
| One-minute temperature | RIPV | 120 | 120 | -0.534 | -1.128 to 0.059 | 0.078 |
| Log TTI | LSPV | 108 | 108 | 0.012 | -0.060 to 0.084 | 0.742 |
| Log TTI | LIPV | 89 | 89 | -0.008 | -0.124 to 0.108 | 0.892 |
| Log TTI | RSPV | 91 | 91 | -0.017 | -0.081 to 0.047 | 0.602 |
| Log TTI | RIPV | 73 | 73 | -0.066 | -0.152 to 0.020 | 0.132 |

Estimates were derived from linear mixed-effects models including OA per 50 mm², OI per 0.1 unit, PV branch, OA-by-branch interaction terms, and a patient-level random intercept. Branch-specific estimates represent the adjusted associations between OA and each cryokinetic outcome within the corresponding PV branch.

CI = confidence interval; LIPV = left inferior pulmonary vein; Log TTI = log-transformed time to isolation; LSPV = left superior pulmonary vein; OA = ostial area; OI = ovality index; PV = pulmonary vein; PVs = pulmonary veins; RIPV = right inferior pulmonary vein; RSPV = right superior pulmonary vein; TTI = time to isolation.
